# Large-Scale Changes in Community Composition: Determining Land Use and Climate Change Signals

**DOI:** 10.1371/journal.pone.0035272

**Published:** 2012-04-16

**Authors:** Christian Kampichler, Chris A. M. van Turnhout, Vincent Devictor, Henk P. van der Jeugd

**Affiliations:** 1 Vogeltrekstation - Dutch Centre for Avian Migration and Demography, NIOO-KNAW, Wageningen, the Netherlands; 2 División de Ciencias Biológicas, Universidad Juárez Autónoma de Tabasco, Villahermosa, Tabasco, Mexico; 3 SOVON Dutch Centre for Field Ornithology, Nijmegen, the Netherlands; 4 Department of Environmental Science and Department of Animal Ecology, Institute for Water and Wetland Research, Radboud University Nijmegen, Nijmegen, the Netherlands; 5 Institut des Sciences de l'Evolution, UMR CNRS-UM2 5554, Université Montpellier 2, Montpellier, France; University of San Diego, United States of America

## Abstract

Human land use and climate change are regarded as the main driving forces of present-day and future species extinction. They may potentially lead to a profound reorganisation of the composition and structure of natural communities throughout the world. However, studies that explicitly investigate both forms of impact—land use and climate change—are uncommon. Here, we quantify community change of Dutch breeding bird communities over the past 25 years using time lag analysis. We evaluate the chronological sequence of the community temperature index (CTI) which reflects community response to temperature increase (increasing CTI indicates an increase in relative abundance of more southerly species), and the temporal trend of the community specialisation index (CSI) which reflects community response to land use change (declining CSI indicates an increase of generalist species). We show that the breeding bird fauna underwent distinct directional change accompanied by significant changes both in CTI and CSI which suggests a causal connection between climate and land use change and bird community change. The assemblages of particular breeding habitats neither changed at the same speed and nor were they equally affected by climate versus land use changes. In the rapidly changing farmland community, CTI and CSI both declined slightly. In contrast, CTI increased in the more slowly changing forest and heath communities, while CSI remained stable. Coastal assemblages experienced both an increase in CTI and a decline in CSI. Wetland birds experienced the fastest community change of all breeding habitat assemblages but neither CTI nor CSI showed a significant trend. Overall, our results suggest that the interaction between climate and land use changes differs between habitats, and that comparing trends in CSI and CTI may be useful in tracking the impact of each determinant.

## Introduction

Among the various anthropogenic factors that are responsible for the extinction of species and the decline of biodiversity, two are considered to have an overwhelming importance, and they have received particular attention in the last few decades: global climatic change [1] and the destruction, fragmentation and disturbance of habitats [2], [3]. Although human land use is regarded to be the main driving force of present-day species extinction, climate change is expected to become at least equally important in the coming decades [4]. The interaction between climate change and habitat loss has been called a “deadly anthropogenic cocktail" [5], and projected future extinction rates are commonly based on the degree of land use and climate change [6]. They will potentially lead to a profound reorganisation of the composition and structure of natural communities throughout the world. However, studies that explicitly integrate both kinds of impact (land use and climate change) are uncommon [7], [8]. Further, biodiversity is simultaneously impacted by changes in climate and land use rather than being separately affected whereby possible effects of the interaction between these two environmental changes remain unexamined [9]. To our knowledge, a formal analysis investigating the long-term change in community composition and dynamics in the face of land use and land cover change (LUCC; see Table 1 for an explanation of all acronyms used in this paper) and climate change is still lacking.

**Table 1:** Acronyms used in this paper.

Acronym	Full wording
BMP	Breeding Bird Monitoring Programme
CSI	Community specialization index
CTI	Community temperature index
LUCC	Land use and land cover change
SSI	Species specialization index
STI	Species temperature index
TLA	Time lag analysis

Any ecological community is experiencing temporal turnover in its composition. Beyond the baseline turnover of natural communities due to stochastic dynamics, current global changes can lead to non-random changes in community composition. To understand the direction and magnitude of global change effects, one must assess whether any observed change in community composition is due to natural change or external factors. In this respect, since climate and land use changes are the main driving forces of community changes, it should be particularly useful to concentrate on the relationship of species to temperature and to habitat specialisation as basic proxies of species susceptibility to these global changes. In this paper we use the breeding bird fauna of the Netherlands as a model assemblage to investigate any potential directional shift in community composition and its relationship with LUCC and increasing temperatures because (i) an extensive national monitoring data set exists which covers abundance estimates of almost 250 species over more than a quarter of a century, and (ii) adequate indices are available to characterise the relationship of bird assemblages with LUCC and temperature change.

To investigate the impacts of global changes on communities, most studies have previously focused on indices which ignore species-specific sensitivity to those changes (e.g., species richness or diversity indices). In such approaches, all species are considered to be equally sensitive to global changes. Ideally, indices accounting for each species-specific response to LUCC or temperature change should improve our ability to assess global change impacts on communities. In this respect, an alternative approach to traditional diversity indices is to first attribute a quantitative trait, *X_i_*, to each species *i* reflecting the specific vulnerability of the species to a given pressure of interest. In doing so, each species can be ranked along a continuous gradient from the least vulnerable to the highest sensitive species for a given pressure (i.e., from the smallest to the largest value of *X_i_*). Then, any given species assemblage at a given point *t* in time can be characterized by averaging trait *X_i_* either across individuals present in this assemblage at that time (if abundances data are available) or simply across species (if only presence-absence data are available). These community level indices are simply a weighted average given by CXI*_t_* = Σ(*a_i_X_i_*)/Σ*a_i_*, where *a_i_* designates the abundances of species *i* in this assemblage and *X_i_* the specific trait of species *i* (note that *a_i_* = 1 for all *i* if only presence-absence data are available). Then, if this assemblage, which is characterised by CXI*_t_* is affected by the given pressure of interest from time *t* to *t*+1, each species should adjust its abundance (or presence) according to its sensitivity to that pressure. This would result in a new value of CXI*_t_*_+1_ which is different from CXI*_t_* and which mirrors the average change of each species-specific response to that pressure in this assemblage. Typically, following an increase of a given pressure to which species are more or less sensitive according to their *X_i_*, species with high *X_i_* should increase relatively faster than those with low *X_i_* so that CXI should increase from t to t+1. Conversely if the pressure decreases, CXI should decrease and remain stable on average if the pressure stays constant.

In practice, this approach was developed to track LUCC impacts on communities using a species-specific level of specialization as a proxy for *X_i_*, the Species Specialization Index, SSI. In brief, as specialists are expected to be replaced by generalists following habitat loss or disturbance, the Community Specialization Index calculated as CSI = Σ(*a_i_*SSI*_i_*)/Σ*a_i_* should decrease following landscape degradation. This index was successfully used at several scales in different habitats and systems [10], [11], [12]. Similarly, this approach was used to quantify climate change impacts on communities. In this case, *X_i_* was replaced by the Species Temperature Index (STI). STI of a given species is simply the average temperature of the species' breeding season range. Following temperature increases, one expects species with breeding areas characterised by high average temperature (i.e., with high STI) to replace those breeding in colder ranges. Therefore, the CTI given by CTI = Σ(*a_i_*STI*_i_*)/Σ*a_i_* is expected to increase following climate warming [13], [14], [15]. Overall, CSI and CTI are two simple ways to quantify whether, and how fast, a given assemblage is affected by land-use and climate change respectively.

Temperature in the Netherlands has increased twice as much as the global average over the past decades [16]. This difference is very unlikely to be due to random fluctuations, but is explained by changes in atmospheric circulation in winter and spring and by changes in soil moisture and cloud cover in spring and summer, processes which are not well simulated in current climate models [17]. Beyond any change to the community composition as a whole, communities occurring in different habitats are expected, however, to respond differently to temperature change. For example, the community of invertebrate-feeding forest birds is assumed to be particularly sensitive since their temporal window of optimal food supply during the breeding season is very narrow and since resource peak and resource demand are strongly synchronised [18]. A mismatch between these two peaks due to phenological changes can lead to strong population declines, notably for long-distance migrants in seasonal habitats such as deciduous forests [19]. The Netherlands are also characterised by continuing high levels of LUCC in the past decades. For example, developed area has increased by 19% from 1981 to 2006, and grassland area has decreased by 25% from 1990 to 2009 [20]. Many species, such as birds that breed in agricultural areas [21], heathland [22], [23] or wetlands [22], [24], [25] are sensitive to land cover change, and changes in community composition might thus be determined by changes in land use and management rather than by temperature change. Effects of interactions between agricultural intensification and temperature change, however, should not be prematurely excluded [26]. Therefore, it is necessary to test whether changes in community composition, CSI and CTI, are similar among habitats. As far as we are aware, the approach of comparing habitat-specific population trends at national scale is quite unique and owes much to the extensive coverage of the Dutch breeding bird monitoring program.

In this paper, we addressed three main objectives. First, we quantified the directional change in composition of Dutch bird communities and determined its essential attributes (no change, white noise, cyclic, directional). Second, we evaluated if a directional community change was present and if so, whether it could be related to temperature change or LUCC. If temperature increase was a driving force of community change, we would observe an increase of the CTI indicating a relative increase of species with a range with high average temperature (those with a higher STI); if LUCC was responsible for community change, we would observe a decrease of the CSI indicating an increasing relative abundance of generalist species (those with a low SSI). Third, we evaluated whether changes in community composition calculated for the entire country (total abundance of each species) differed among habitats, namely forest, heath, dunes and coast, wetlands and farmland.

## Materials and Methods

### Data origin

**Figure 1:**
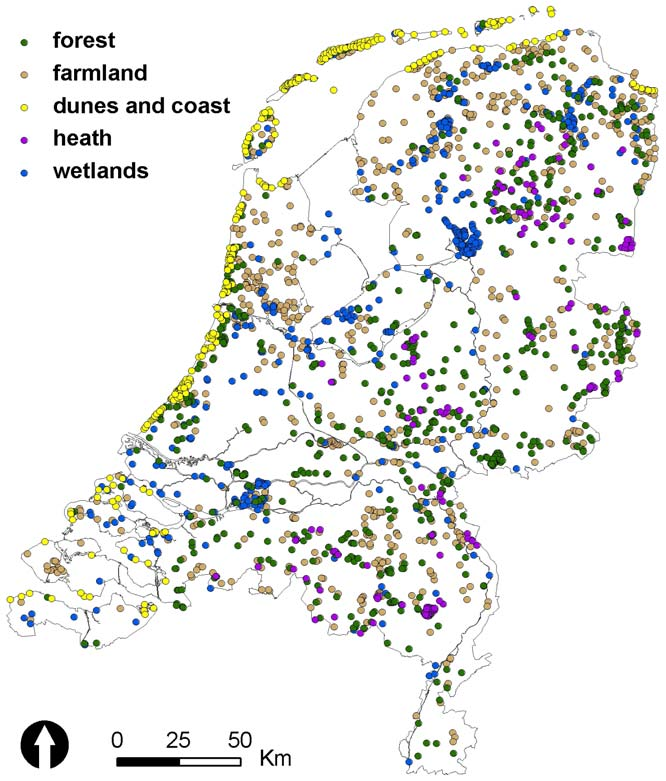
Locations of the study plots of the Dutch Breeding Bird Monitoring Programme.

Data were drawn from the Dutch Breeding Bird Monitoring Programme (BMP) and date from 1984 to 2009. The BMP is based on territory mapping [27] in fixed study plots throughout the Netherlands (Figure 1). All common and scarce breeding birds in the Netherlands were covered (S = 244). Fieldwork and interpretation methods were highly standardised and described in detail in a manual by van Dijk [28]. Territory mapping uses a high and annually constant number of field visits (5–10 between March and July). Size of study plots, as well as the exact number, timing and duration of visits, depend on habitat type and species selection. For this study, only those study plots were included in which all breeding bird species were counted. The number of study plots varied from 293 at the beginning of the programme in 1984 to a maximum of 957 (in 2003) (Figure S1). We assumed that the varying number of study plots did not affect the patterns of relative abundance in the data, perhaps with the exception of a few very rare species whose detection probability might have been lower in the years with less study plots. All birds with territory-indicative behaviour (e.g. song, pair bond, display, alarm and nests) were noted on field maps. Species-specific interpretation criteria were used to determine the number of “territories" per species at the end of the season. Interpretation criteria focussed on the type of behaviour observed, the number of observations required (taking into account the varying detection probability between species and within the breeding season), and the period of observations (to exclude non-breeding migrants). Detection probability was not explicitly quantified in the BMP. The applied interpretation criteria, however, are based on a study of species-specific detection probabilities. Thus, for species with a high detection probability a larger number of observations is required to accept a territory than for species with a low detection probability [28]. We considered the number of “territories" to be a proxy of true abundance and expect approximate linear relationships between the surveyed samples and the total population size of each species [23].

### Time-lag analysis

Time-lag analysis (TLA) was introduced by Collins *et al.* [29] and is a distance-based approach used to study temporal dynamics of ecological communities by regressing community dissimilarity over increasing time lags (one-year lags, two-year lags, three-year lags, etc.). To prevent the smaller number of data points of larger time lags from biasing the result, the time lags are square-root transformed. If the slope of the regression line of dissimilarity on lag is positive, this implies that the community is undergoing directional change. Directional change can also be caused by stochastic variation as long as the abundance trajectories of the constituent species are first-order Markov chains (i.e., abundance at time *t* of a given species is dependent on its abundance at time *t*−1) (Kampichler & van der Jeugd, submitted). If the regression line is negative, a convergent dynamics of the community is inferred, i.e., the community returns to an earlier state of the time series such as following perturbation or of other cyclical behaviour. In cases where a community is composed of species governed by white noise dynamics (i.e., abundance at time *t* is independent from abundance at any time *t*−1, *t*−2, … ) or composed of species with constant means, the slope of the regression line is not significantly different from zero; in all other instances the slope is significant, even when the temporal change is very slow. Generally, they range between 0.02 and 0.25, dependent on the proportions of species with different dynamics and on their temporal variability; higher proportions of species with constant means imply shallower slopes; higher proportions of species with stochastic dynamics or directional change imply steeper slopes. We transformed all abundance data according to the Hellinger transformation, as proposed by Legendre and Gallagher [30], *N′_ij_* = sqrt(*N_ij_*/Σ*N_+j_*), where *N_ij_* is the population size of species *i* in year *j*, and Σ*N_+j_* is the sum of individuals across all species in year *j*. Hellinger distance (i.e., Euclidean distance of Hellinger transformed data) has the advantage of making assemblages directly comparable independent of their species richness and the abundance of their constituent species. As a consequence, TLA based on transformed data is sensitive only to changes of relative abundance patterns but not to mere increase or decrease (Kampichler & van der Jeugd, submitted).

Since a time series of *n* years yields (*n* ^2^−*n*)/2 distance values (*n*−1 values for lag 1, *n*−2 values for lag 2, …, one value for lag *n*−1), the number of degrees of freedom is heavily inflated and the data points are not independent, which prohibits the determination of statistical significance based on the variance. Significance of the TLA slopes was thus determined by a Monte Carlo permutation [31]. We randomly reordered the year columns in the species x year data matrices (10,000 permutations), and determined the error probability *p* by dividing the number of random slopes that were steeper than the observed slope by the number of permutations. Differences between slopes of communities in different habitats (see below) were also compared by a resampling approach: we used a bootstrap sample (with replacement) from the original species list of a community to generate a new species x year matrix with untransformed data and applied the same procedure to the community to be compared. We Hellinger transformed both matrices and determined the difference of their TLA slopes. Bootstrapping was repeated 10,000 times, and the smaller proportion of random slope differences that was larger or smaller than zero, respectively, was multiplied by 2 to determine statistical significance of the slope difference [32].

### Community temperature index

STI—the long-term average temperature experienced by the individuals of a species over its range in the breeding season—was determined for each species using distributional data from the European Bird Census Council atlas of European breeding birds [33] as well as patterns of mean annual temperatures across Europe from the WorldClim database (URL http://www.worldclim.org) [13]. For any given assemblage, the CTI was calculated by averaging the STI of the constituent species weighed by their relative abundances [13]. Note that, although for each species only a part of its range was considered, the CTI based on European climate data can be applied due to the high correlation between continentally and regionally determined STI [13]. To estimate the temporal trend in CTI, we modelled yearly change in CTI across the studied sites using site as a random factor, year as a continuous covariate, and an exponential spatial autocorrelation structure. This model provided us with the temporal trend in CTI accounting for spatial autocorrelation and for between site variations in the level of CTI.A positive slope in the temporal trend in CTI would indicate a local increase in individuals with high STIs. Species with higher STIs are those breeding in “hotter" ranges on average. Since these species are also those breeding in southern latitudes, an increase in CTI can be viewed as reflecting the replacement of northerly distributed species by southern species.

### Community specialisation index

SSI—the degree of habitat specialization—was measured for each species as the variance of average densities among the different habitat classes applying the same habitat classification for all species. If a given species was absent in a given habitat, this was accounted for in the calculation of SSI. The coefficient of variation was used as the metric since it is statistically independent of the average species density [34]. For a detailed discussion on the calculation of SSI see [35]. We used the SSI values as calculated by van Turnhout *et al.* [23], which are based on the variation in abundance of species in 12 different habitats in the Netherlands, using abundance data from BMP study plots in 2001–2005. Habitats were classified using aerial photography and were calibrated in the field by field-workers. We calculated the CSI of the complete Dutch bird fauna by averaging the SSI of the constituent species weighed by their abundance. To estimate the temporal trend in CSI we used the same statistical framework as the one used for CTI. A positive slope would indicate a relative increase of species with high SSI, i.e., species that are restricted to one or few given habitats (specialists), whereas a negative slope would indicate a relative increase of species that are able to exploit various types of habitats (generalists). The SSI and STI of the Dutch breeding bird species are uncorrelated (*r_S_* = 0.030, *p* = 0.714, Figure S2), thus the probability for a confounding effect between these two classification systems is very low.

All analyses were conducted separately for the entire dataset and for study plots located in specific habitats. In this latter case, we used the following habitats: forests (deciduous, coniferous and mixed woodlands; parks), dunes and coasts (coastal dunes, saltmarshes and beaches), heaths (heathlands and inland drift sands), wetlands (open water and freshwater marshlands) and farmlands (grasslands and arable land). The number of study plots was insufficient to include urban habitats. For all statistical analyses the significance level was *p*<0.05.

## Results

**Figure 2:**
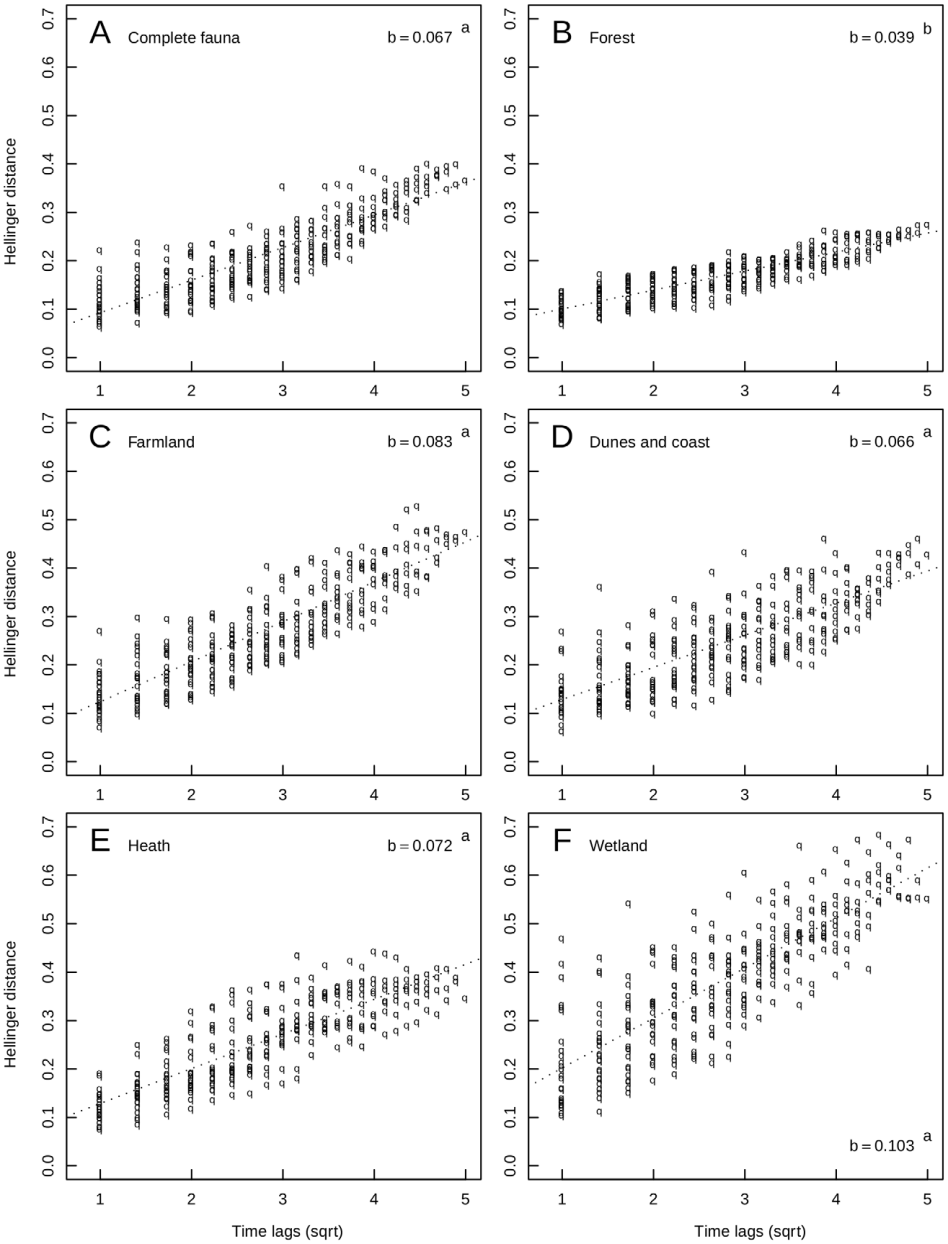
Time lag analysis of Hellinger transformed Dutch bird abundances for the complete breeding bird fauna (A), and for breeding bird communities in forests (B), farmland (C), dunes and coast (D), heath (E) and wetland (F) between 1984 and 2009. Dotted lines represent the linear regressions of Hellinger distance on square root transformed time lag. The respective slopes, *b*, are reported within each panel. All *p* are <0.0001. Slopes sharing superscripts are not different at *p*<0.05.

The Dutch breeding bird fauna, as well as all habitat communities underwent highly significant directional change (*p*<0.0001) between 1984 and 2009 (Figure 2). The magnitude of directional change in the time series differed between the habitat communities. The wetlands community displayed the most drastic change (TLA slope *b* = 0.103) (Figure 2F), followed by the farmland (*b* = 0.083) (Figure 2C) and the heath community (b = 0.072) (Figure 2E). However, these slopes are not statistically different from the total trend (*b* = 0.067) (Figure 2A). The slope of the community on coast and dunes was almost identical to the total trend (*b* = 0.066) (Figure 2D). The least amount of change was found in the forest community (*b* = 0.039) (Figure 2b) which was significantly lower than in the complete bird fauna and in any of the other assemblages.

**Figure 3:**
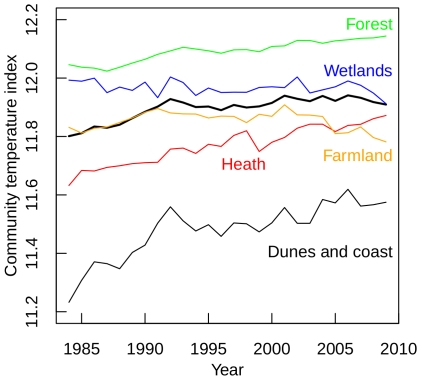
Temporal trend of the community temperature index of the entire Dutch breeding bird fauna (bold line) and for breeding bird communities in forests, farmland, dunes and coast, heath and wetland between 1984 and 2009.

The CTI of the complete Dutch bird fauna over time had a significant positive slope (*b* = 0.0037) (Figure 3, Table 2) indicating a relative increase of species with hotter breeding ranges. Among the communities in different breeding habitats, the dunes and coast, the heath and the forest birds (in decreasing order) showed significant positive slopes, while the farmland community showed a significant negative slope. No significant trend in CTI could be identified for the wetlands community. All but a few CTI trends were significantly different from each other (pairwise ANCOVA) (Table 2).

**Table 2:** Temporal trends of the community temperature index (CTI) and community specialisation index (CSI) of Dutch birds from 1984 to 2009.

		CTI				CSI			
Bird community	df	Slope	S.E.	*F*_1,df_	*p*	Slope	S.E.	*F*_1,df_	*p*
Complete fauna	15748	3.7*10^−3 a^	3.2*10^−4^	137.5	****	−1.8*10^−3 a^	2.2*10^−4^	65.87	****
Dunes and coast	3762	1.0*10^−2 b^	9.4*10^−4^	103.0	****	−4.9*10^−3 b^	5.6*10^−4^	77.12	****
Heath	888	7.9*10^−3 b^	6.7*10^−4^	136.5	****	1.4*10^−3 c^	1.1*10^−3^	1.72	0.190
Forest	3111	4.4*10^−3 a^	2.7*10^−4^	266.5	****	−4.1*10^−5 c^	2.0*10^−4^	0.04	0.833
Wetlands	2163	−5.8*10^−4 c^	6.8*10^−4^	0.7	0.394	−7.6*10^−4 ac^	5.1*10^−4^	2.21	0.151
Farmland	5233	−2.2*10^−3 c^	4.6*10^−4^	22.0	****	−7.1*10^−4 ac^	3.3*10^−4^	4.62	0.032

Slopes of a given index sharing a superscript are not different at p<0.05.

**** , p<0.0001.

**Figure 4:**
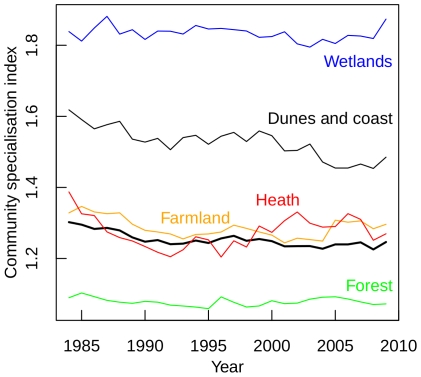
Temporal trend of the community specialisation index of the entire Dutch breeding bird fauna (bold line) and for breeding bird communities in forests, farmland, dunes and coast, heath and wetland between 1984 and 2009.

The complete Dutch bird fauna was characterised by a significantly negative trend of the CSI over time (*b* = −0.0018) (Figure 4, Table 2) indicating a relative increase of generalist species. The steepest negative trend was displayed by the dunes and coast community (*b* = −0.0049). The CSI trend in dunes and coast was significantly different from CSI trends elsewhere (pairwise ANCOVA). The CSI trends for wetland, forest and heath communities were not significant (Table 2).

## Discussion

### Directional change in Dutch bird fauna

We showed that the Dutch breeding bird fauna underwent distinct directional change between 1984 and 2009. However, to accurately evaluate how much this change resulted from anthropogenic impact on the environment, rather than reflecting natural processes, it would be desirable to have baseline data of temporal changes in ecological communities against which anthropogenic impact can be measured [36], [37]. Unfortunately, such data is meagre since community oriented long-term research on breeding birds has been conducted primarily on local forests (for example, [38]–[43]). Even if communities were locally undisturbed, they would be affected by large scale consequences of human activities, for example, acid rain, nitrogen deposition or global temperature change. The only meaningful comparison of our results can thus be with data from large remote pristine ecosystems where natural turnover has the lowest possible anthropogenic influence. There are long-term breeding bird data sets available from c. 300 km^2^ of subalpine vegetation in Sweden [44] and c. 1500 km^2^ of temperate primeval forest in Poland and Belarus [43]. TLA slopes of these studies average 0.031 (Sweden) and 0.035 (Poland) (Kampichler *et al.* in preparation) and are much lower than the slopes of the Dutch breeding birds with exception of the Dutch forest bird assemblage which shows a slope (*b* = 0.039) almost as low. Although the community dynamics in study plots with typically less than 1 km^2^ cannot be directly compared with the dynamics of a national fauna, we cautiously interpret that species turnover in the Netherlands is faster than would be expected in an assemblage with only natural turnover and that it has been accelerated by human activities. For example, the fast change observed in communities from Dutch wetlands is indicative of (i) the consequences of decades of habitat destruction and disturbance, (ii) the subsequent recovery of many wetland species after the implementation of wetland conservation measures from the 1990s onwards and (iii) the colonisation of the Netherlands by waterfowl species as a result of legal protection and increased plant food quality due to changes in agricultural practice [22], [23], [25], [45]–[47]. Its slope of 0.103 is almost as high as the TLA slopes of successional temperate forests on abandoned fields (b = 0.125, data from [38]) or after clear-cutting (*b* = 0.106, data from [48]). These represent the steepest slopes that have been observed in all available long-term data sets on breeding bird communities (Kampichler et al. in preparation).

### Effects of temperature change and land use change

The significant changes in both CTI and CSI indicate that the fast changes in community composition during the last 25 years are related to both temperature change and land use change. Interestingly, changes in CTI and CSI differ among habitats indicating that their breeding bird assemblages are not equally affected by temperature change versus LUCC.

In farmlands, both CTI and CSI declined significantly. However, these trends were very weak compared to the other significant trends (Figure 3, see F-values in Table 2) and are probably primarily driven by the high statistical power yielded by the large dataset used, rather than by profound ecological responses. Moreover, the negative trend in CTI appeared far from significant if calculated for the period 1984–2007, i.e. after removing the last two years in the time series (*b* = −0.000458, *p* = 0.35). Indeed, the direction of CTI change in farmland was contrary to CTI change in the other habitats, and also contrary to our expectation as based on literature [26]. However, we did expect a strong and negative change in CSI. Population declines are observed for a large number of farmland specialists in the Netherlands and abroad. This is thought to be related to the intensification of agricultural practice across Europe [49], [50] which has reduced nesting and feeding opportunities and breeding success in meadow birds, while agri-environment schemes have not yet resulted in favourable effects for these species [51], [52]. Furthermore, the openness of the unique Dutch polder landscape in the western part of the country has decreased as a result of the establishment of young forestry plantations and urban expansion. This has resulted in an increasing simplification of ecosystems through loss of specialist species and an increase of a large number of widespread shrub and woodland generalists [22]. Remarkably, these ongoing processes were not reflected very clearly from changes in CSI, although the decrease in CSI is quite consistent and substantially stronger for the period 1984–2004 (*F*_1,4090_ = 135, *b* = −0.0047, *p*<0.0001, Figure 4).

In forests and heathlands, CTI increased while CSI remained stable. At first sight, one might attribute this to the wide-spread declines of long-distance migratory species which in the Netherlands include several northerly species, such as the Pied Flycatcher *Ficedula hypoleuca*, the Willow Warbler *Phylloscopus trochilus* and the Wood Warbler *P. sibilatrix*. Their decline is most probably caused by an increasing mismatch between timing of food requirements and food availability as a result of spring warming, and the decline is most severe in habitats with a seasonal food peak, such as forests [19], [53]. Also regarding the heathlands, evidence shows that food availability for breeding birds in spring has a sharper peak compared to other habitats [54]. A closer look, however, reveals that the shift towards a higher CTI in the forest and heath communities was mainly caused by an increase in species with a relatively high STI (centre of distribution in an area *warmer* then the Netherlands; increasing 9, equal 1, decreasing 3), and not so much by a decrease in species with a relatively low STI (centre of distribution in an area *colder* then the Netherlands; increasing 7, equal 8, decreasing 9). The area and quality of forests increased during the past decades, since forests matured and became more attractive for hole-nesting breeding birds and raptors [22]. This increase was probably enhanced by changes in forest management [55]. However, since the change in CSI was not significant, this implies that generalists benefited to the same extent as forest specialists at the community level.

The stable CSIs in forests and heaths do not support the results of a previous study which concluded that forest specialists were thriving, whereas heathland specialists were declining at a faster rate than generalists [23]. However, that study focused on changes at the species level instead of the community level, and special emphasis had been given to very rare and localised species that were poorly represented in the Dutch BMP study plots used for the present study. In particular, the encroachment of heath by nitrophilic grasses and shrubs, which has replaced the original low vegetation that was characterised by a large fraction of bare ground [56], has limited nesting and foraging conditions for specialists of early successional habitats [23]. The same processes occurred in dunes [57] and have negatively affected populations of specialists in coastal habitats [22], which—in contrast to heathlands—are well reflected in a decline of CSI.

Although the assemblage of wetland birds underwent the most pronounced community change over time (as suggested by TLA), there is no indication that the temperature change or LUCC have affected community patterns. In this assemblage, the combined effects of habitat destruction and subsequent conservation measures, as well as the increase of many herbivorous waterfowl as outlined above, most probably overrule the ‘simple’ effects of disturbance and climate.

### Conclusions

**Figure 5:**
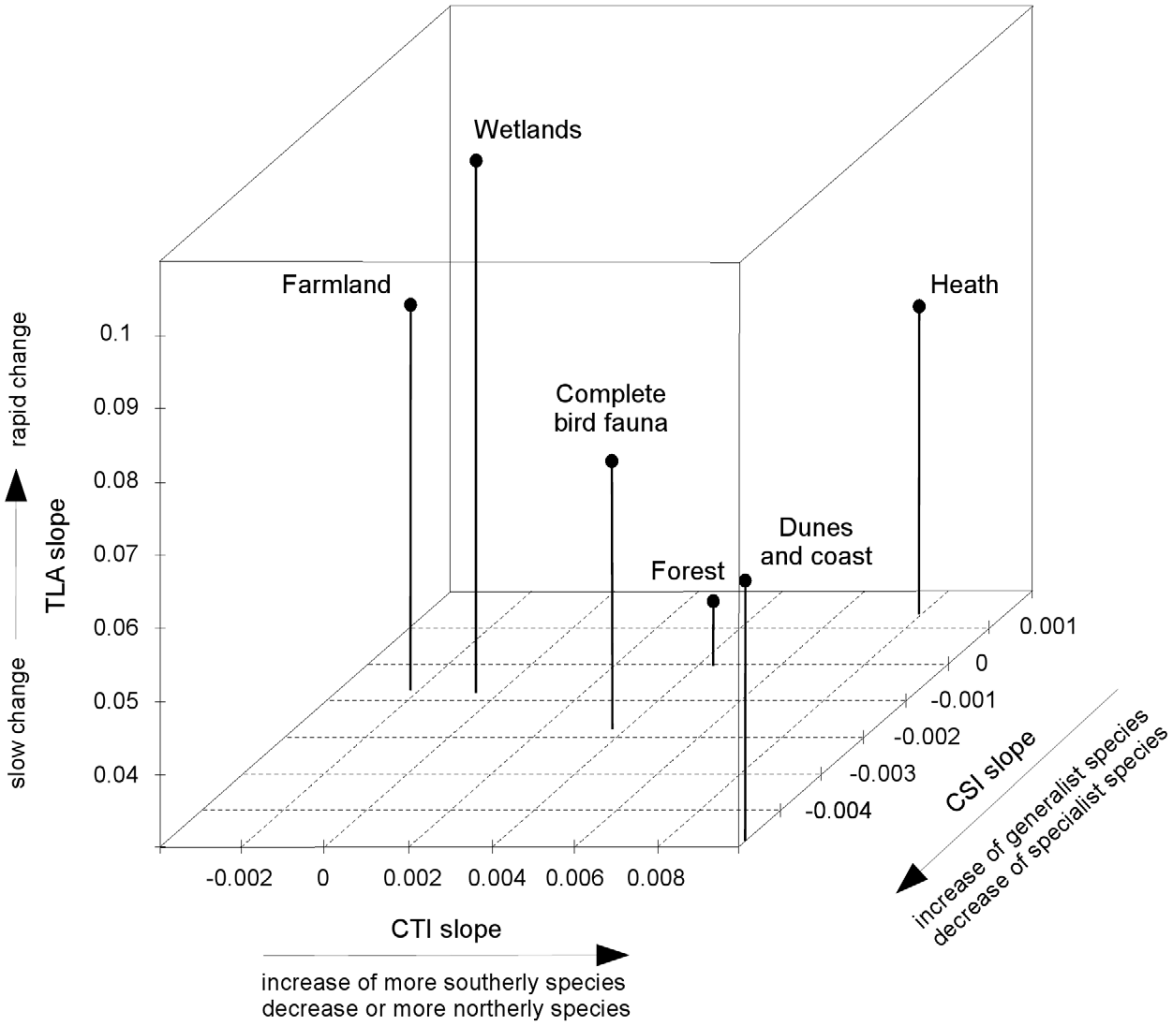
Comparison of the slope of time lag analysis (TLA) and the temporal trends of the community temperature index (CTI) and the community specialisation index (CSI) of the entire Dutch breeding bird fauna and breeding bird communities in forests, farmland, dunes and coast, heath and wetland.

The Dutch breeding bird fauna has experienced a distinct change of community composition in the past 25 years which is accompanied by a significant land use signal and an even more significant climate signal. Our results convincingly show that assemblages of particular breeding habitats are not equally affected by climate versus land use changes, and that studies focussing on a subset of species inhabiting one or more particular habitats might not be valid to judge the effects of climate on changes in population trends. Habitat communities differ in velocity of community change and the relative importance of LUCC and temperature change, and they hold very different positions in variable space defined by these factors (Figure 5).

Since the interaction between LUCC and temperature change or the decoupling between them is context dependent, the study of the combination of trends in TLA/CSI/CTI among assemblages in different land use systems (or in different habitats, or in protected versus non-protected areas, or between any regions of interest) may help to highlight which assemblages are more sensitive to one or the other pressure. We conclude that the combined application of TLA, CTI and CSI is a valuable approach for determining the effects of land use change and temperature change on natural communities.

## Supporting Information

Figure S1 **Number of study plots of the Dutch Breeding Bird Monitoring Programme per year.**
(TIF)

Figure S2 **Relationship between species temperature index (STI) and species specialisation index (SSI) of Dutch breeding birds.**
(TIF)
