# Supplementary figures and images for: Large-Scale Changes in Community Composition: Determining Land Use and Climate Change Signals
Source: PLoS One. 2012 Apr 16;7(4):e35272. doi: 10.1371/journal.pone.0035272 (PMC3327650; doi:10.1371/journal.pone.0035272)

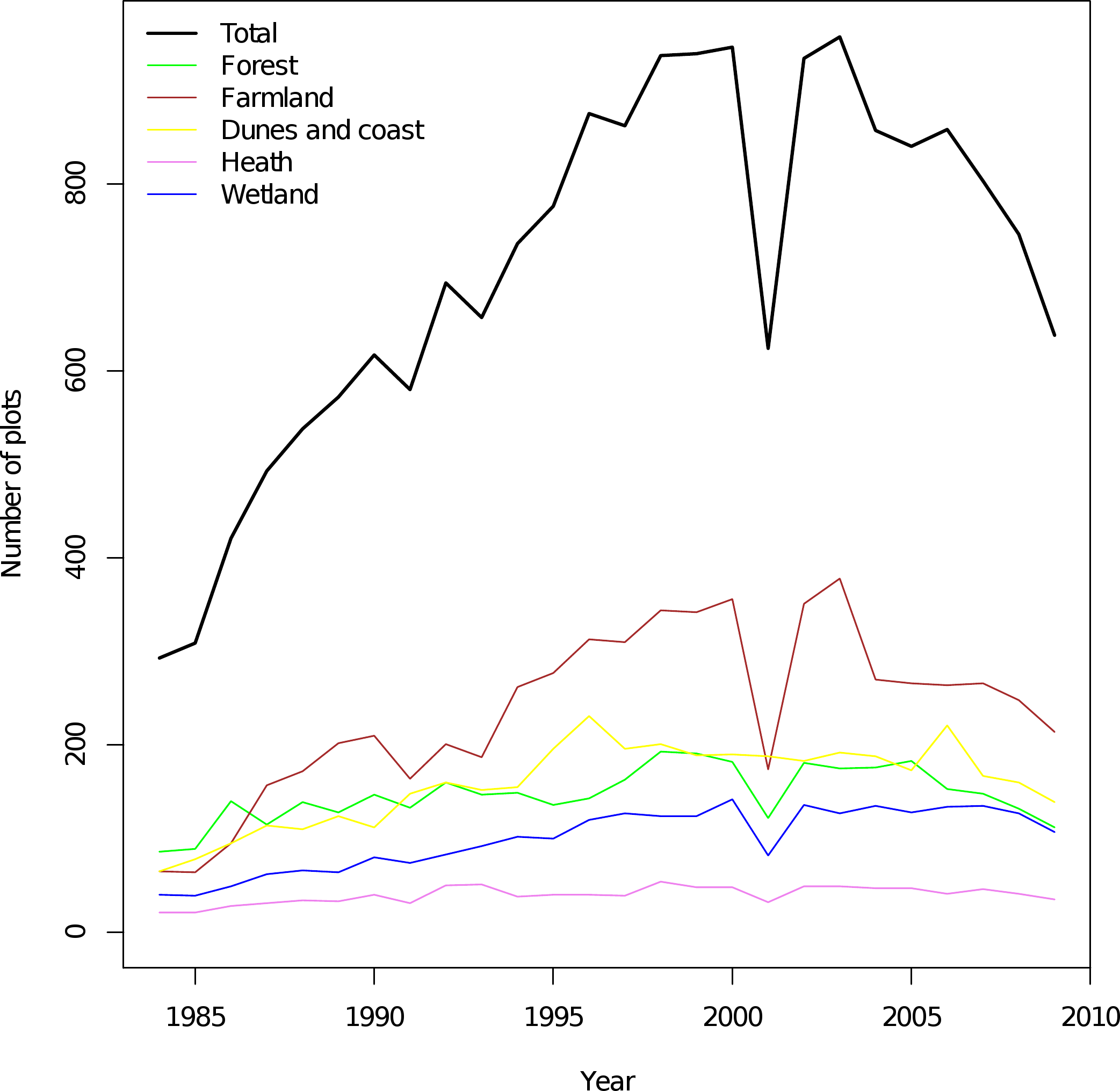

Supplement: Figure S1 — Number of study plots of the Dutch Breeding Bird Monitoring Programme per year. (TIF) [file pone.0035272.s001.tif]

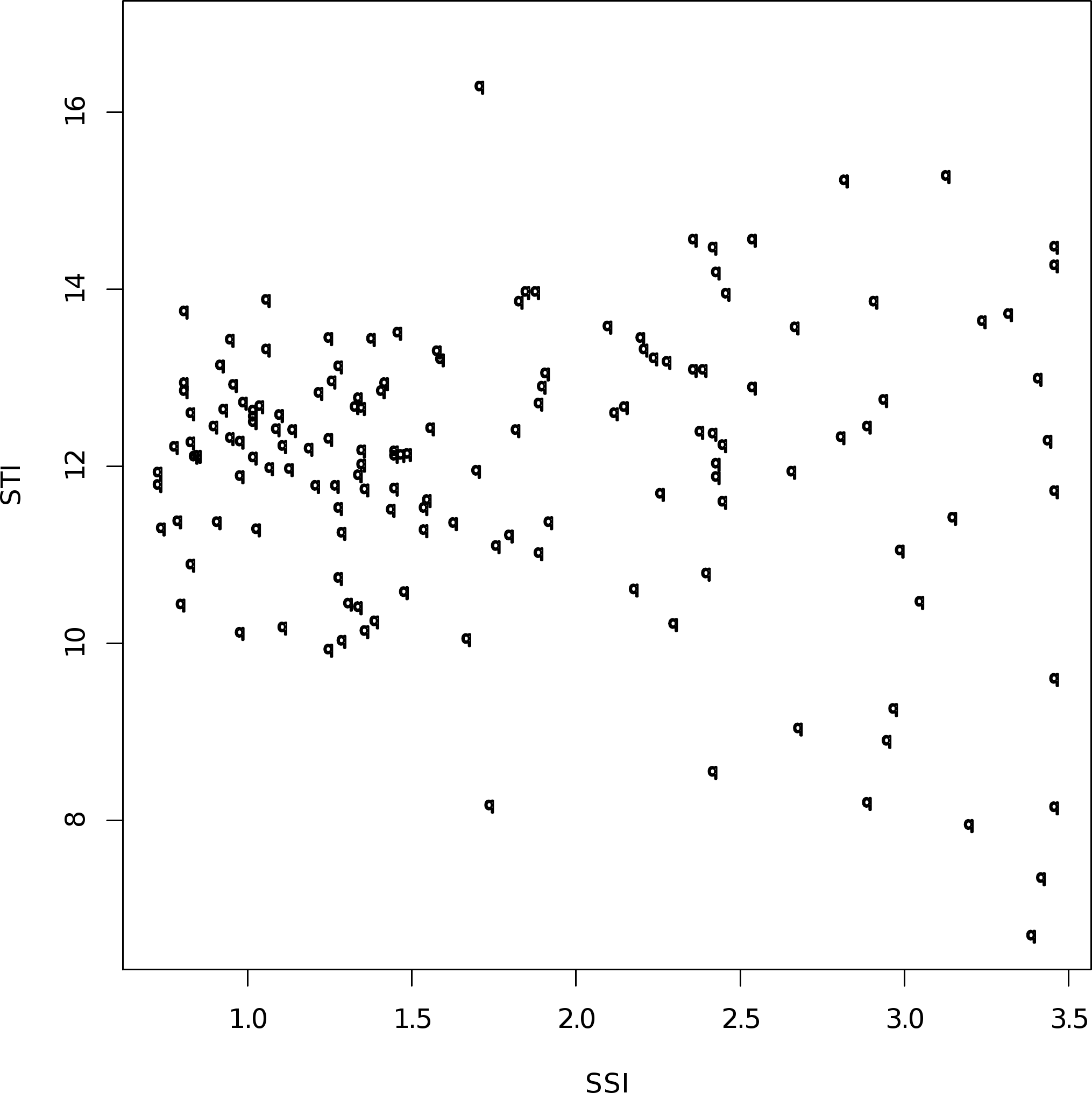

Supplement: Figure S2 — Relationship between species temperature index (STI) and species specialisation index (SSI) of Dutch breeding birds. (TIF) [file pone.0035272.s002.tif]
